# Rare presentation of Pseudomonas aeruginosa meningitis following intrathecal pump replacement

**DOI:** 10.1016/j.inpm.2025.100548

**Published:** 2025-02-13

**Authors:** Cyrus Ghaffari, Rajiv Reddy, Tim Furnish

**Affiliations:** Center for Pain Medicine, University of California San Diego, La Jolla, CA, United States

To the Editor,

We report a rare case of Pseudomonas aeruginosa meningitis following intrathecal pump (IP) replacement. A 69-year-old male with a history of spastic diplegia cerebral palsy, T11-S1 spinal fusion, and chronic low back pain presented six weeks after an uneventful IP replacement. His symptoms included the worst headache of his life, neck pain, and chills. The pump, originally implanted in 2006 for spasticity management, had been revised due to battery expiration. On examination, the surgical site appeared normal, and an initial lumbar puncture (LP) was unremarkable. Despite empiric antibiotics, his condition deteriorated with worsening mental status and seizures. Subsequent cerebrospinal fluid (CSF) side port aspiration and pump pocket lavage identified Pseudomonas aeruginosa. Following pump explantation and targeted meropenem therapy, the patient's condition improved significantly. We received patient consent to publish details of their case.

This case underscores the importance of early CSF sampling before initiating antibiotics in suspected infections and highlights the potential for meningitis to occur despite a normal-appearing pump pocket.

The patient presented to the emergency department six weeks postoperatively with new symptoms. Imaging, including CT and MRI, showed no evidence of abscess or fluid collection. Despite the normal appearance of the pump site, he developed hallucinations and seizures. An LP was completed by interventional radiology due to his extensive spinal fusion. Unfortunately, this LP was delayed by a week due to scheduling issues but was ultimately unremarkable. Empiric antibiotics were started due to the clinical suspicion of infection. An MRI of the brain on hospital day seven showed findings concerning for ventriculitis and borderline leptomeningeal enhancement. Despite a negative initial LP, the patient's mental status continued to worsen. Next, side port and pump pocket aspiration were completed. There was no fluid in the pump pocket to aspirate, so saline was injected into the pocket then aspirated to be sent for culture. The pocket lavage demonstrated a positive Gram stain, while the CSF sample from the side port was concerning for meningitis with ∼270 WBCs (69 % neutrophils), low glucose, and slightly elevated protein. Later, the cultures grew Pseudomonas.

At the time of pump replacement surgery, intraoperative cefazolin was administered intravenously and vancomycin powder was placed into the pump pocket. The patient was given a five-day postoperative course of cephalexin after surgery. Upon hospital presentation with suspected meningitis, empiric antibiotics were initiated. Despite antibiotic therapy his condition worsened. Following device removal and appropriate antibiotic therapy, the patient's condition improved dramatically.

Infections associated with intrathecal pumps are serious, with rates reported between 3.2 and 27.5 % in the literature [1]. Infection rates seem to be higher in patients receiving IT therapy for spasticity versus for chronic pain [1]. Infections of intrathecal pumps may arise from the surgical procedure, pump refills, or indirectly through hematogenous spread. According to the 2017 Infectious Diseases Society of America's Clinical Practice Guidelines for Healthcare-Associated Ventriculitis and Meningitis, it is often difficult to distinguish meningitis from local infections related to infusion pumps [2]. In one retrospective review referenced in the guidelines, 3 out of 26 patients with an infection complication developed meningitis alongside an incisional infection, while 4 had meningitis without incisional infection [3]. In our case, the pump pocket showed no obvious signs of infection either on physical examination or intraoperatively during the pump explantation. Our differential also included Froin syndrome, as well as the possibility of contaminated baclofen. Froin syndrome describes a CSF sample with a high protein level, xanthochromia, and hypercoagulability due to meningeal irritation and/or CSF blockage by a tumor or mass. This could explain why the MRI brain showing signs of meningitis and LP were not consistent. However, other imaging studies did not show any CSF blockages. It is also possible that the initial CSF sample from the lumbar puncture did not show an infection picture due to the patient having been treated with antibiotics for a week prior to the LP. A second MRI brain during his hospitalization showed evidence of “debris” in the ventricles with the differential including contaminated baclofen which could result in a chemical meningitis. Of note, the FDA released a warning in 2018 of potential contamination of a baclofen active pharmaceutical ingredient from Taizhou Xinyou Pharmaceutical & Chemical Co.

Pseudomonas infections are particularly concerning due to high mortality rates and antibiotic resistance. Intrathecal antibiotic delivery has been proposed as a salvage strategy when pump removal poses significant risks, although evidence remains limited [4]. In this case, systemic antibiotic therapy combined with pump removal resulted in a favorable outcome.

This case emphasizes the importance of thorough investigation in suspected pump infections, even when the surgical site appears normal. Clinicians should consider early side port CSF sampling and prompt pump removal in cases of suspected Pseudomonas infection. Further research into intrathecal antibiotic therapy as a salvage option is warranted.

## Declaration of competing interest

None.
